# Cysteine-Rich Intestinal Protein 1 Served as an Epithelial Ovarian Cancer Marker via Promoting Wnt/*β*-Catenin-Mediated EMT and Tumour Metastasis

**DOI:** 10.1155/2021/3566749

**Published:** 2021-08-06

**Authors:** Yujuan Liu, Wenyu Li, Ji Luo, Yiguo Wu, Yuanyuan Xu, Tingtao Chen, Wei Zhang, Fen Fu

**Affiliations:** ^1^Department of Gynaecology and Obstetrics, The Second Affiliated Hospital of Nanchang University, No. 1 Mingde Road, Nanchang 330006, China; ^2^Department of Gynaecology and Obstetrics, The First Hospital of Nanchang, No. 128 Xiangshan North Road, Nanchang 330006, China; ^3^Queen Mary School, Nanchang University, No. 999 Xuefu Avenue, Nanchang 330031, China; ^4^Institute of Translational Medicine, Nanchang University, No. 999 Xuefu Avenue, Nanchang 330031, China

## Abstract

**Objective:**

To explore the expression, functions, and the possible mechanisms of cysteine-rich intestinal protein 1 (CRIP1) in epithelial ovarian cancer.

**Methods:**

Using open microarray datasets from The Cancer Genome Atlas (TCGA), we identified the tumorigenic genes in ovarian cancer. Then, we detected CRIP1 expression in 26 pairs of epithelial ovarian cancer tissue samples by immunohistochemistry (IHC) and performed a correlation analysis between CRIP1 and the clinicopathological features. In addition, epithelial ovarian cancer cell lines A2780 and OVCAR3 were used to examine CRIP1 expression by western blot and qRT-PCR. Various cell function experiments related to tumorigenesis were performed including the CCK8 assay, EdU, Annexin V-FITC/PI apoptosis assay, wound healing, and Transwell assay. In addition, the expression of epithelial-mesenchymal transition (EMT) markers was detected by western blot to illustrate the relationship between CRIP1 and EMT. Furthermore, KEGG pathway enrichment analysis and western blot were conducted to reveal the signaling pathways in which CRIP1 is involved in ovarian cancer pathogenesis.

**Results:**

CRIP1 was identified as an oncogene from the TCGA database. The IHC score demonstrated that the CRIP1 protein was expressed at a higher level in tumours than in tumour-adjacent tissues and was associated with a higher pathological stage, grade, and positive lymphatic metastasis. In cell models, CRIP1 was overexpressed in serous epithelial ovarian cancer. Cell function experiments showed that the knockdown of CRIP1 did not significantly affect cell proliferation or apoptosis but could exert an inhibitory effect on cell migration and invasion, and also induce changes in EMT markers. Furthermore, KEGG pathway enrichment analysis and western blot showed that CRIP1 could induce ovarian cancer cell metastasis through activation of the Wnt/*β*-catenin pathway.

**Conclusion:**

This study is the first to demonstrate that CRIP1 acts as an oncogene and may promote tumour metastasis by regulating the EMT-related Wnt/*β*-catenin signaling pathway, suggesting that CRIP1 may be an important biomarker for ovarian cancer metastasis and progression.

## 1. Introduction

Ovarian cancer, a type of malignancy in the ovary, is the most lethal gynaecological carcinoma as well as the fifth leading cause of cancer mortality among women [1]. Over 85% of ovarian cancers originate from the epithelium (known as epithelial ovarian cancer, EOC), with serous ovarian cancer being the most common histological subtype [2]. Currently, the main treatment options for epithelial ovarian cancer are tumour cell reduction surgery and platinum-based chemotherapy, immunotherapy, and targeted therapy. Although these therapeutic options are available, the overall five-year survival rate remains low, at around 30%-40% [2]. It is estimated that 22,530 patients were diagnosed with ovarian cancer in 2019 in the United States, of which 13,980 patients died [3]. The high mortality may be due to a number of factors, including the lack of symptoms and detectable biomarkers for ovarian cancer in its early stage, distant invasion, and metastasis [4, 5]. Thus, it is of great importance to identify new predictive biomarkers for early diagnosis and have a better understanding of the mechanism of ovarian cancer metastasis, which is also an urgent issue for improving the efficacy of ovarian cancer treatment.

There are many factors contributing to the metastasis of ovarian cancer. Recently, accumulated evidence has indicated that a major driver of ovarian cancer metastasis is epithelial-mesenchymal transition (EMT), in which cancer cells lose their epithelial potential and acquire a mesenchymal phenotype, allowing cells to detach from the primary site and gain the ability to metastasise [6–9]. In addition, research has shown that the activity of EMT is regulated by many different signaling pathways [10], among which the canonical Wnt/*β*-catenin signaling pathway is pivotal for both ovarian carcinogenesis and EMT. Sun and colleagues [11] found that the high expression of Golgi phosphoprotein 3 (GOLPH3) in ovarian cancer patients could be regarded as an oncogene and was related to poor prognosis, as it could promote ovarian cancer cell proliferation, migration, and invasion by stimulating the Wnt/*β*-catenin signaling pathway and EMT. Dong et al. [12] also reported that PEST-containing nuclear protein (PCNP) promoted the ability of ovarian cancer cells to invade and metastasise in the same way. These findings not only highlight the significance of the Wnt/*β*-catenin signaling pathway in carcinogenesis and EMT but also revealed that some oncogenes are involved in ovarian cancer invasion and metastasis. This has enriched our knowledge of the mechanisms of ovarian cancer progression and provided a basis for cancer therapy, which has become more precise and individualised. However, the information in this field is still not complete. Due to the small number of studies on this topic, more in-depth research is necessary to further reveal the exact mechanisms of ovarian cancer invasion and metastasis and further improve current treatment strategies.

As a member of the LIM/double zinc finger protein family, cysteine-rich intestinal protein 1 (CRIP1) has a unique double zinc finger motif and is primarily expressed in the intestine [13]. It was first recognised as an intracellular zinc transport and absorption protein [14]. CRIP was also detected in immune cells such as peritoneal macrophages and peripheral blood mononuclear cells, suggesting it may be involved in host immune responses [15]. Moreover, in recent decades, the aberrant expression of CRIP1 in several cancers has attracted increasing attention. In metastatic colorectal cancer (CRC), CRIP1 was overexpressed and downregulation of CRIP1 was found to inhibit cell migration and invasion in the cell lines SW620 and HT29 [16]. Thus, it may function as an oncogene to regulate the migration and invasion of CRC cells and may be regarded as a new promising biomarker for poor prognosis and the metastasis of colon cancer. Similar results were also found in cervical cancer [17], thyroid carcinoma [18], and endometrial cancer [19]. In contrast, CRIP1 expression led to a favourable outcome and fewer metastases in osteosarcoma [20] and breast cancer [21]. Although the role of CRIP1 seems to be controversial in the abovementioned tumours, we are certain that CRIP1, as an oncogene or tumour suppressor gene, has a close relationship with tumour metastasis. However, the current studies of CRIP1 are finite and the underlying mechanisms of CRIP1-mediated tumour metastasis are largely unknown. Moreover, the relationship between ovarian cancer and CRIP1 has not yet been discussed.

In this study, our objective was to describe CRIP1 expression patterns, functions, and possible mechanisms in ovarian cancer. First, we used bioinformatics methods to screen out the oncogene CRIP1 in ovarian cancer from the TCGA database, showing that ovarian cancer with the high expression of CRIP1 had a poor prognosis. Subsequently, we used tissue samples and cell models to verify its expression. Then, we performed *in vitro* experiments to explore its function in invasion, migration, and EMT and further uncovered the possible mechanisms. The results show that the upregulation of CRIP1 can promote ovarian cancer invasion, metastasis, and EMT by activating the Wnt signaling pathway, suggesting that CRIP1 could be viewed as a valuable new biomarker for ovarian cancer.

## 2. Materials and Methods

### 2.1. Genetic Screening

Using the online differential gene expression analysis tool (http://gepia2.cancer-pku.cn/#degenes), we analysed the differential gene expression of ovarian cancer and normal ovarian tissues from The Cancer Genome Atlas (TCGA) database. Differentially expressed genes were determined by means of ANOVA. Genes with log_2_ FC cut − off > 1 were considered upregulated, and *P* < 0.05 were considered statistically significant. Then, GDC TCGA Ovarian Cancer (OV) mRNA expression FPKM data and clinical survival data were downloaded from the website (https://xenabrowser.net/datapages/), and significant survival genes were obtained through the “Survival” and “SurvMiner” packages of the R software (*P* < 0.05). By further searching on PubMed, genes reported in the literature were no longer considered. After taking the intersection of the three, the target genes were obtained.

Subsequently, the target genes were ranked by -log_10_ (*P* value) to show the top five genes which were used for the analysis of survival and hazard ratio (HR) on the survival analysis website (https://kmplot.com/analysis/). HRs were applied to identify different genes, with HR < 1 indicating protective genes and HR > 1 indicating risk genes. The genes that were both significantly associated with overall survival and HR > 1 were identified as key genes. Finally, we analysed the relationship between key genes and ovarian cancer by using the online gene expression DIY tool (http://gepia2.cancer-pku.cn/#degenes).

### 2.2. Kyoto Encyclopaedia of Genes and Genomes (KEGG) Pathway Enrichment Analysis

The median expression value of the key gene in ovarian cancer samples from the TCGA database was selected as an optimal cut-off value to classify different subgroups which had high or low expression and were then used for KEGG analysis. The R software “Limma” package was used to analyse gene expression levels in cancer samples, and the differentially expressed genes were then screened out. Pearson correlation analysis between CRIP1 and differentially expressed genes was carried out using R language, and the differentially related genes were selected with the condition of *P* < 0.01. We conducted KEGG pathway enrichment analysis of these differentially related genes online (http://www.webgestalt.org/).

### 2.3. Patients and Tissue Samples

Paraffin-embedded tissue samples (cancer tissue and paracancerous tissue) were collected from patients at the Second Affiliated Hospital of Nanchang University, with 50 serous ovarian cancer samples and 25 paracancerous tissue samples. The patients were diagnosed with serous ovarian cancer by surgical pathology. The basic data of patients were collected, including age, lesion size, clinicopathological grading, staging, lymphatic metastases, and preoperative CA125 levels. Patients who received chemotherapy, immunotherapy, or hormone therapy before surgery or those with ovarian cancer combined with other cancers were excluded. Ethical approval was granted by the Ethical Committee of the Second Affiliated Hospital of Nanchang University.

### 2.4. Cell Lines and Cell Culture

The normal ovarian cell line IOSE80 and human epithelial ovarian cancer cell lines OVCAR3 and A2780 were obtained from Shanghai EK-Bioscience Biotechnology Co., Ltd. A2780 and IOSE80 cell lines were incubated in RPMI-1640 medium containing 10% foetal bovine serum (FBS) and 1% penicillin-streptomycin (PS), while OVCAR3 in RPMI-1640 medium supplemented with 20% FBS, 1% insulin, and 1% PS. Cells were grown in an incubator at 37°C with 5% CO_2_. When the cell confluence was about 80-90%, the cells in good condition were collected and digested with trypsin for subculture or frozen for later experiments.

### 2.5. Immunohistochemistry

All tissue samples were paraffin-embedded and cut into 3-5 mm sections, which were heated in an oven at 72°C for 2 hours, followed by xylene deparaffinization and ethanol gradient rehydration. To block and inactivate endogenous peroxidase, the sections were maintained in 3% H_2_O_2_ at ambient temperature for 15 minutes, followed by washes in phosphate-buffered saline (PBS). After boiling in citrate buffer and cooling naturally, the sections were again washed with PBS three times for antigen retrieval. Then, the sections were incubated with reagent A (normal goat serum) and anti-CRIP1 rabbit polyclonal antibody (Abcepta, Cat No. AP4707b). The slides were washed with PBS and then incubated with biotin-labelled secondary antigens and rinsed again, followed by streptavidin labelled with horseradish peroxidase for another round of incubation. DAB-H_2_O_2_ was used as the chromogenic reagent for visualisation, and haematoxylin was added for counterstaining. Then, the software ImageJ was applied to calculate grey values for pathological scoring.

A semiquantitative method was used to detect the expression grade of CRIP1 protein. Percentages of positive cells (0%, 1-25%, 26-50%, 51-75%, and 76-100%) were recorded as 0, 1, 2, 3, and 4 points, respectively. Positive staining intensity was scored as follows: colourless (0 point), pale yellow (1 point), brownish yellow (2 points), and dark brown (3 points). The expression grade was determined by multiplying the two scores: 0-5 represented low expression and 6-12 represented high expression.

### 2.6. Western Blot

Following the standard procedure, we extracted protein from the OVCAR3, A2780, and IOSE80 cell samples and then measured the protein content with the BCA protein assay kit. After SDS-PAGE electrophoresis, the membrane was transferred and blocked and then incubated with primary and secondary antibodies. The protein content was analysed by X-ray exposure. The antibodies used in this experiment were as follows: *β*-actin (Cat No. 4970S), *β*-catenin (Cat No. 8084S), MMP-2 (Cat No. 87809S), and MMP-9 (Cat No. 13667S) were bought from Cell Signaling Technology (Danvers, MA, USA); CRIP1 (Cat No. 15349-1-AP), E-cadherin (Cat No. 20874-1-AP), N-cadherin (Cat No. 22018-1-AP), GSK-3*β* (Cat No. 22104-1-AP), and p-GSK-3*β* (Cat No. 14850-1-AP) were purchased from Proteintech (Wuhan, China); vimentin (Cat No. bs-8533R) was obtained from Bioss (Beijing, China).

### 2.7. Quantitative Real-Time Reverse Transcription PCR

Following the manufacturer's protocol, we isolated total RNA from OVCAR3 cells with TRIzol reagent and reverse-transcribed it into cDNA using a reverse transcription kit (TaKaRa, China). Real-time quantitative PCR was performed on a fluorescent PCR instrument using SYBR Green PCR master mix kits (TaKaRa, China). GAPDH was chosen as the internal control. The relative fold relationship was calculated using fold change = 2^−ΔΔCt^. After repeating the experiments three times, the mean value was calculated. All primers were purchased from General Biol (Anhui, China).  Table 1 shows the specific primer sequences.

### 2.8. siRNA Interference

Three siRNA oligonucleotides targeting the CRIP1 gene, including si-168, si-229, and si-276, were designed and synthesised by General Biosystems (Anhui, China). The three siRNA oligonucleotide sequences are listed as follows: si-168: 5′-GCAACAAGGAGGUGUACUUTT-3′; si-276: 5′-ACGCUGAGCACGAAGGCAATT-3′; and si-229: 5′-CUGCCUGAAGUGCGAGAAATT-3′. OVCAR3 cells were placed at a density of 1 × 10^5^ cells/ml into 6-well plates. When the cells were at 30-50% confluent, we conducted transfection. The OVCAR3 cells were transfected with 20 *μ*M small interfering RNA (siRNA) or negative control siRNA (si-NC) (General Biosystems Anhui, China) in reduced serum medium using Lipofectamine™ 3000 Transfection Reagent (Thermo Fisher, USA). After transfecting for 24 to 48 h, the effect of gene knockdown was measured by western blot and qRT-PCR.

### 2.9. Wound Healing Assay

Cells were seeded on 6-well plates and incubated for 24 hours or longer until cells reached 80-90% confluence, before a scratch wound was created by drawing a straight line with a 10 *μ*l pipette tip. Then, the cells were rinsed with PBS three times and placed in serum-free medium, followed by incubation in an incubator at 37°C with 5% CO_2_. Images were taken at 0 and 24 hours. Wound closure was quantified by randomly selecting under the microscope and measuring the remaining unmigrated area using ImageJ in three different fields at each time point. Wound healing percentage (%) = (Area of original wound − Area of actual wound)/Area of original wound × 100%.

### 2.10. Transwell Assay

After digestion and suspension in serum-free medium, cells were adjusted to a density of 5 × 10^5^/ml. The cell suspension (100 *μ*l) was seeded into the upper Transwell chambers, and the lower chambers were filled with 500 *μ*l of 20% FBS medium. For invasion experiments, the Transwell chambers were Matrigel-coated. Then, the cells were incubated at 37°C for 24 hours in order to assess migration or invasion. After 4% paraformaldehyde fixation and haematoxylin staining of the lower surface, the cells were counted under a microscope.

### 2.11. 5-Ethynyl-20-Deoxyuridine (EdU) Incorporation Assay

Cell proliferation was evaluated using an EdU Imaging kit (US Everbright Inc., Suzhou, China). Cells were placed into 24-well plates at 5 × 10^3^ cells per well for 24 hours, followed by exposure at 1× EdU (10 *μ*M) at 37°C for 2 hours. The cells were then fixed with 4% paraformaldehyde and permeabilised with 0.5% Triton X-100. After treatment with 100 *μ*l of dyeing reaction cocktail for 30 minutes, the cells were incubated with 300 *μ*l of 1× Hoechst (5 *μ*g/ml) for 20-30 minutes. Finally, EdU-stained cells were visualised under a fluorescence microscope (Olympus). According to the formula (EdU − positive cells/Hoechst − stained cells) × 100%, we calculated the percentage of EdU-positive cells by arbitrarily selecting three fields in each group.

### 2.12. Cell Counting Kit-8 (CCK8) Assay

We used a Cell Counting Kit-8 (GlpBio, USA) to measure cell viability. OVCAR3 cells were cultured in a 96-well plate with 100 *μ*l culture medium at a concentration of 5 × 10^3^ cells/well, and the dish was then placed into an incubator for preincubation (37°C, 5% CO_2_). After 24 hours of incubation, 10 *μ*l of transfected OVCAR3 cells was added to the dish and incubated for 1 to 4 days. Then, 10 *μ*l of CCK8 solution was added to each well, and the dish was placed in the incubator again for 1-4 hours. The solution was mixed gently on the orbital shaker for 1 minute to ensure the colour was distributed evenly. The OD_450_ were measured with a microplate reader.

### 2.13. Annexin V-FITC/PI Apoptosis Assay

Apoptosis was examined using an Annexin V-FITC Apoptosis Detection kit (Dojindo, Japan) following the manufacturer's instructions and relevant reference [22]. Cells were cultured in 6-well plates overnight. After treating with or without siRNA interference for 24 h, cells were trypsinised with EDTA-free trypsin, followed by three washes with cold PBS. Then, the cells were resuspended in 1× binding buffer to reach a final concentration of 1 × 10^6^ cells/ml. Next, 100 *μ*l of the above cell suspension was collected and mixed with 5 *μ*l of Annexin V-FITC and 5 *μ*l of PI working solution for 15 minutes at room temperature in the dark. Images were then taken using an inverted fluorescence microscope (Olympus).

### 2.14. Statistical Analysis

All results are presented as the mean ± standard deviation. Student's *t*-test and one-way ANOVA were used to determine statistical significance, and Pearson's chi-squared (*χ*^2^) test was used to identify the significance of correlations between the expression of CRIP1 and histopathological factors. All data were analysed using GraphPad Prism 7 software, and *P* < 0.05 was considered significant.

## 3. Results

### 3.1. Key Genes Identified by Bioinformatics in Ovarian Cancer

**Figure 1:**
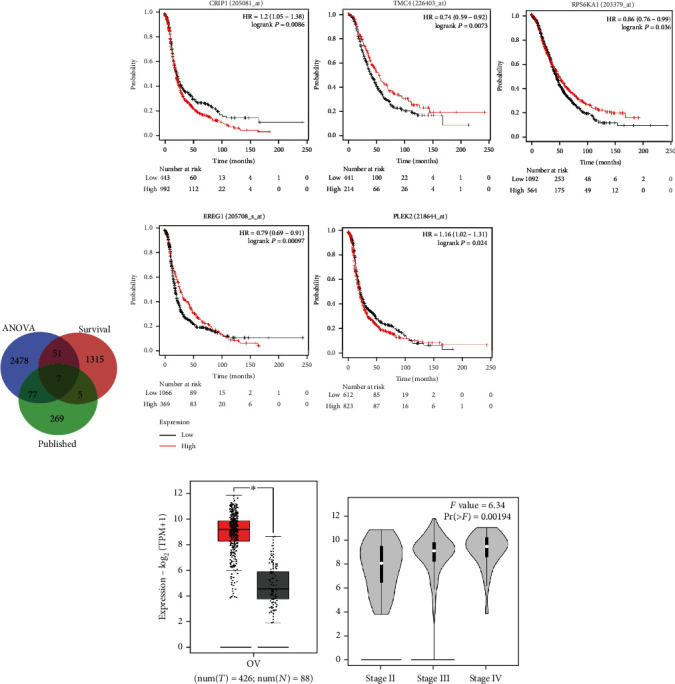
Genetic screening and bioinformatics analysis of cysteine-rich intestinal protein 1 (CRIP1). (a) 2,613 genes were significantly upregulated in ovarian cancer tissues using the online differential gene expression analysis tool, and 1,378 survival significant genes were obtained through survival analysis; 358 genes were excluded for being published on PubMed. After taking the intersection of the three, 51 target genes were obtained in the end. (b) After ranking the 51 target genes by -log_10_ (*P* value), the top five genes were obtained, and then, the survival and hazard ratio (HR) analysis of these genes was conducted on the survival analysis website. The survival plots of the top five target genes suggested that only high expression of CRIP1 and PLEK2 could affect prognosis (*P* < 0.05). (c) Box plot showing the significantly upregulated expression of CRIP1 in ovarian cancer tissues (left, red; *n* = 426) compared with nonneoplastic tissues (right, black; *n* = 88) (*P* < 0.05). (d) Violin plot showing a correlation between increased CRIP1 expression and increased pathological grade in ovarian cancer (*P* < 0.05).

To determine the key genes responsible for ovarian cancer, we used the TCGA dataset and online tools. The results show that 2,613 genes were significantly overexpressed in ovarian cancer tissues compared with normal ovarian tissues in the TCGA database and 1,378 survival significant genes were obtained through survival analyses. Meanwhile, 358 genes that were reported in the literature were no longer considered. After taking the intersection of the three, 51 target genes were obtained in the end (Figure 1(a)), which represent not only differential genes but also significant survival genes. To further identify the key genes, we ranked 51 target genes by -log_10_ (*P* value). Finally, the top five key genes were found (Table 2). Next, the top five genes were used for the analysis of survival and HR on the survival analysis website to reveal their oncogenic properties. The survival plots suggested that the key genes significantly associated with overall survival and HR > 1 were CRIP1 and PLEK2 (Figure 1(b)). Because CRIP1 was more statistically significant and associated with a higher risk, it was chosen for the following study.

Next, we demonstrated CRIP1 expression in ovarian cancer and its relationship with disease. The box plot shows the significantly upregulated expression of CRIP1 in ovarian cancer tissues compared with adjacent nonneoplastic tissues (∣log_2_ FC∣ cut-off: 1, *P* value; cut-off: 0.01, *P* < 0.05) (Figure 1(c)). Interestingly, the violin plot shows that CRIP1 expression increased with an increase in tumour pathological stage, suggesting a close positive correlation between CRIP1 expression and ovarian cancer grade (*F* = 6.34, *P* < 0.05) (Figure 1(d)). These findings indicate that the CRIP1 gene has oncogenic properties, and the high expression of CRIP1 could be a reliable indicator of poor prognosis in ovarian cancer.

### 3.2. CRIP1 Is Overexpressed in Ovarian Cancer Cell Lines and Cancer Tissues

**Figure 2:**
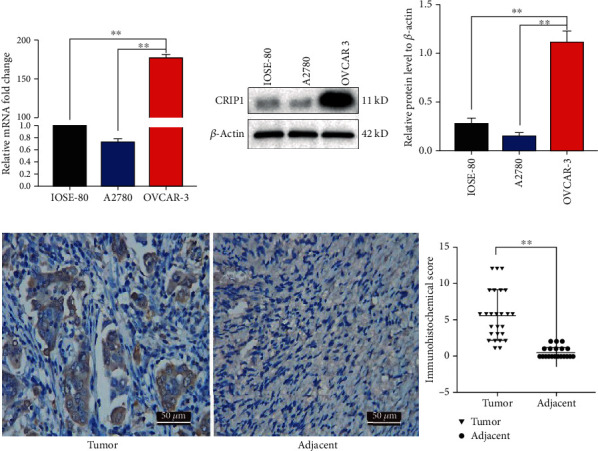
CRIP1 is highly expressed in both ovarian cancer tissue samples and cell lines. (a, b) The mRNA levels (a) and protein levels (b) of CRIP1 in the normal ovarian cell line IOSE-80 and epithelial ovarian cancer cell lines OVCAR3 and A2780 were detected by qRT-PCR and western blot. (c) Immunohistochemical detection of CRIP1 protein levels in tissue samples (cancerous and adjacent tissues) (left). CRIP1 protein is higher in ovarian cancer tissues than tumour-adjacent normal tissues of the same patient (right).

To verify the expression of CRIP1 in ovarian cancer, we selected two ovarian cancer cell lines (A2780 and OVCAR3) and used the normal ovarian cell line IOSE80 as the control. When compared with IOSE80, the mRNA level of CRIP1 was significantly upregulated in OVCAR3 (*P* < 0.01), whereas CRIP1 expression was relatively unchanged in A2780 (*P* = 0.9350, Figure 2(a)). The western blot also showed the same result at the protein level (Figure 2(b)). Furthermore, the CRIP1 content in ovarian cancer tissues was detected by immunohistochemistry (IHC), including 26 pairs of serous epithelial ovarian cancer tissues with matched adjacent normal tissues. IHC score showed that CRIP1 was highly expressed in cancer tissues, in contrast to no expression in adjacent normal tissues (*P* < 0.01, Figure 2(c)). This result was consistent with the results of the bioinformatics analysis.

In addition, a correlation analysis between the CRIP1 expression and the clinicopathological parameters in 50 cases of serous epithelial ovarian cancer was conducted. Our data showed that high CRIP1 expression was closely related to higher pathological stage, grade, and positive lymphatic metastasis, whereas no relation was detected with age, tumour diameter, or CA125 level (Table 3), indicating that CRIP1 may promote ovarian cancer aggressiveness and distant metastasis. Given the experimental results above, the OVCAR3 cell line was selected for subsequent experiments.

### 3.3. si-CRIP1 Has No Significant Effect on Cell Proliferation and Apoptosis in OVCAR3 Cells

**Figure 3:**
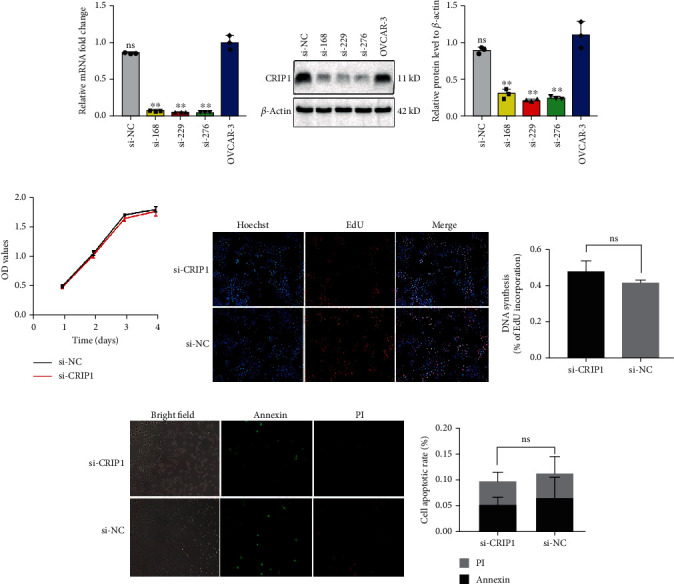
Short interfering RNAs (siRNAs) were used to deplete CRIP1 gene expression *in vitro*. The effect of si-CRIP1 in the OVCAR3 cell line showed no difference in cell proliferation and apoptosis. (a, b) qRT-PCR (a) and western blot (b) were used to detect the gene silencing effect of OVCAR3 cell lines transfected with si-CRIP1 or si-NC. GAPDH and *β*-actin were used as internal controls. (c) The influence of CRIP1 silencing on cell viability in the OVCAR3 cell line. After transfection with si-CRIP1 or si-NC for 1 day, 2 days, 3 days, and 4 days, cell viability was assessed using CCK8, and OD_450_ was measured. (d, e) The influence of CRIP1 silencing on cell proliferation (d) and apoptosis (e) in the OVCAR3 cell line. After transfection with si-CRIP1 or si-NC, cell proliferation and apoptosis were assessed by the EdU and Annexin V-FITC/PI assays, respectively. Representative graphs are shown on the left. Mean ± SD represents the data of three independent experiments. ns: no statistical significance. ^∗^*P* < 0.05 and ^∗∗^*P* < 0.01.

We confirmed a high level of expression of CRIP1 in OVCAR3 and ovarian cancer tissues. To determine the role of CRIP1 in ovarian cancer, we knocked down CRIP1 in OVCAR3 cells using three different short interfering RNAs (siRNAs), including si-168, si-229, and si-276. Transfection efficiency was verified by qRT-PCR and western blot (Figures 3(a) and 3(b)). Among these three siRNAs, si-229 showed the best silencing effect, so it was chosen for the following experiments.

Next, we evaluated CRIP1 effects on proliferation and apoptosis with or without CRIP1 siRNA. The results from CCK8 assays revealed that CRIP1 silencing did not affect cell viability (*P* > 0.05, Figure 3(c)). Similarly, the EdU assay suggested that CRIP1 knockdown had no significant effect on cell proliferation (*P* = 0.2589, Figure 3(d)). Furthermore, using the Annexin V-FITC/PI assay to detect apoptosis, we found that there was no difference between si-CRIP1 transfected cells and si-NC transfected cells, suggesting that si-CRIP1 in OVCAR3 cells had no effect on apoptosis (*P* = 0.7637, Figure 3(e)).

### 3.4. CRIP1 Silencing Abates Migration and Invasion in OVCAR3 Cells

**Figure 4:**
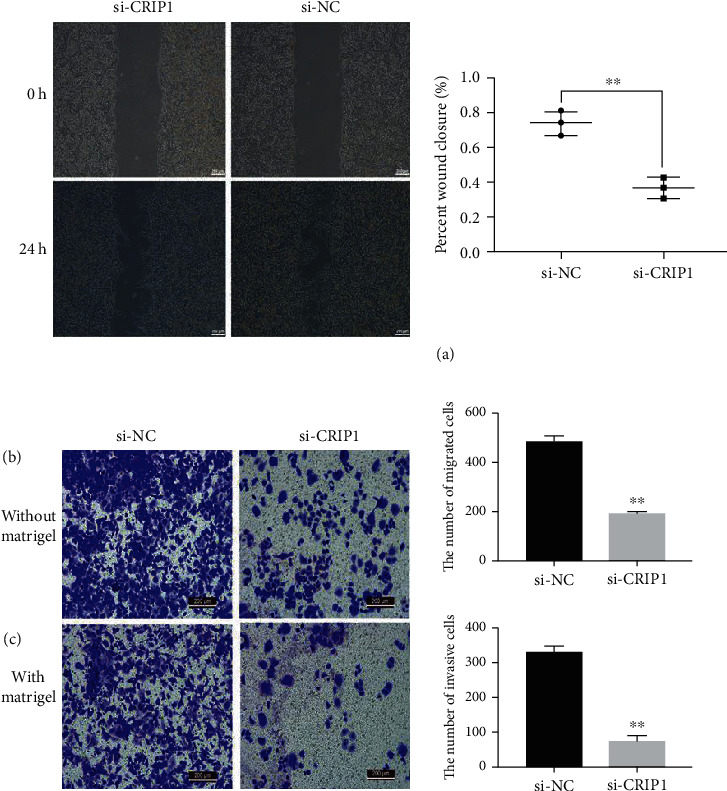
The depletion of the CRIP1 gene inhibited cell migration and invasion in the OVCAR3 cell line. (a) Wound healing assay and (b) Transwell assays without and with (c) Matrigel were used to detect cancer cell migration and invasion abilities, respectively. Representative graphs are shown on the left.

The wound healing assay was conducted to demonstrate whether CRIP1 silencing affects OVCAR3 cell migration. Compared with the si-NC group, the cells with si-CRIP1 showed a more extensive wound closure area (Figure 4(a)), which suggested a significant decrease in migration ability in si-CRIP1 OVCAR3 cells (*P* < 0.01). Similar results were also found in the Transwell assay without Matrigel (*P* < 0.01, Figure 4(b)). These results demonstrate that CRIP1 silencing inhibited cell migration. To understand the role of CRIP1 in cancer cell invasion, we carried out another Transwell assay with Matrigel. As shown in Figure 4(c), when compared with the si-NC group, fewer cells in the si-CRIP1 group entered the lower chamber through the Matrigel-coated membrane (*P* < 0.01). These results revealed that the depletion of CRIP1 suppresses migration and invasion in OVCAR3 cells.

### 3.5. CRIP1 Induces EMT by the Wnt/*β*-Catenin Pathway

**Figure 5:**
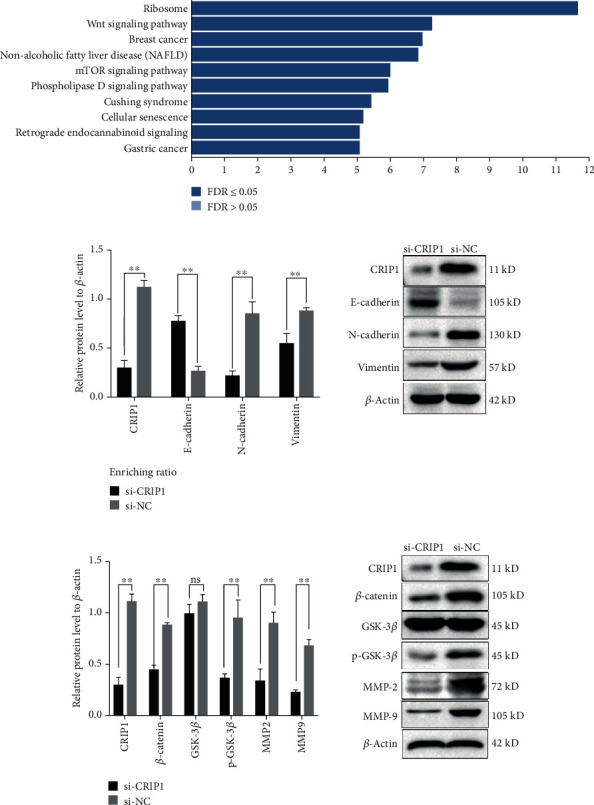
The relationship between CRIP1 and EMT indicates that CRIP1 may function in ovarian cancer development via the Wnt/*β*-catenin pathway. (a) KEGG pathway enrichment analysis was performed to identify which pathway was significantly enriched in CRIP1-related ovarian cancer progression. (b) The effect of CRIP1 silencing on EMT-related signaling pathways. EMT markers, such as E-cadherin, N-cadherin, and vimentin proteins, were detected by western blot. (c) The effect of CRIP1 silencing on Wnt/*β*-catenin signaling pathway. The important molecules of this pathway, including *β*-catenin, GSK-3*β*, p-GSK-3*β*, MMP2, and MMP9, were assessed by western blot.

The above results suggested a clear correlation between CRIP1 expression and cell invasion. As EMT plays a critical role in the process of migration and invasion, it is necessary to further verify the relationship between CRIP1 and EMT. Western blot was carried out to detect the expression of EMT markers, including E-cadherin, N-cadherin, and vimentin. After silencing the CRIP1 gene using CRIP1 siRNA, we observed that the epithelial marker E-cadherin was upregulated, and the mesenchymal markers N-cadherin and vimentin were downregulated (Figure 5(b)). Combined with the results of cell function experiments, we deduced that CRIP1 is involved in the process of cell migration and invasion by regulating EMT in ovarian cancer.

To explore the biological pathways by which CRIP1 is involved in ovarian cancer pathogenesis, we further conducted KEGG pathway enrichment analysis. The results show that the Wnt/*β*-catenin signaling pathway was significantly enriched (Figure 5(a)). As the Wnt/*β*-catenin signaling pathway is vital to EMT and cancer metastasis, we selected this pathway for further study and investigated the key proteins of the canonical Wnt/*β*-catenin pathway by western blot. Our results demonstrate that *β*-catenin, p-GSK-3*β*, and downstream genes including matrix metalloproteinase (MMP-2 and MMP-9) were markedly downregulated after CRIP1 silencing, while GSK-3*β* was unchanged (Figure 5(c)). In general, these results indicate that CRIP1 regulates EMT by modulating the Wnt/*β*-catenin pathway.

## 4. Discussion

Ovarian cancer is a gynaecological malignancy, and its high mortality rate has been shown to be largely caused by the metastatic nature of the cancer. Thus, finding a new predictive biomarker for early diagnosis and targeted therapy is of great importance. Although many genetic alterations have been shown to play a part in ovarian cancer metastasis, the potential mechanism is still elusive. Recently, CRIP1, with only one LIM domain, has been studied in several malignancies including colorectal cancer, breast cancer, cervical cancer, and osteosarcoma [20, 21, 23, 24]. It can induce different outcomes in different cancer types, acting as either a tumour-specific suppressor gene or an oncogene [20, 25]. However, its role has not yet been identified in ovarian cancer.

Here, we revealed the function of CRIP1 in ovarian cancer and its underlying mechanism for the first time. Through analysis of the TCGA database, we confirmed that CRIP1 was upregulated in serous ovarian cancer samples and was closely associated with tumour progression. Then, we used tissue samples and cell models to confirm these results. We observed the same trend; i.e., that CRIP1 had a markedly higher expression level in ovarian cancer tissues than in normal samples, suggesting an oncogenic property of CRIP1. However, it is interesting to observe that this result was only seen in the serous ovarian cancer cell line OVCAR3, while CRIP1 expression was relatively unchanged in A2780 cells when compared with normal cells. The reason for this may be that A2780 is a mucinous epithelial ovarian cancer cell line that has a different genetic background, and that CRIP1 expression has a close relationship with tumour type. Further research revealed that elevated CRIP1 was closely correlated with a higher pathological stage, grade, and positive lymphatic metastasis of patients, pointing to a link between increased CRIP1 expression and ovarian cancer aggressiveness. Taken together, these findings show that CRIP1 expression is closely related to tumour type, and that as an oncogene, CRIP1 can serve as a biomarker for a specific diagnosis and as an indicator of early metastasis in serous epithelial ovarian cancer patients.

To date, the role of CRIP1 in tumours has been controversial. To further investigate its oncogenic properties, we silenced the expression of CRIP1 and performed *in vitro* experiments. The results showed no significant change in proliferation and apoptosis in ovarian cancer cells, which was consistent with a recently published study on CRIP1-depleted colorectal cancer cells [16]. However, this result does not agree with a study on breast cancer in which CRIP1 acted as an inhibitor of proliferation and invasion processes [21]. This confirms the previous statement that CRIP1 function may differ depending upon cancer type. Moreover, the wound healing and Transwell assays showed that CRIP1 was mainly involved in the tumour metastasis process.

Next, we explored the mechanism of CRIP1 in regulating tumour metastasis. As EMT is a major driver of cancer metastasis [26], we further verified the relationship between CRIP1 and EMT. As the results show, CRIP1 knockdown induced the increased expression of the epithelial marker E-cadherin and decreased the expression of the mesenchymal markers N-cadherin and vimentin. E-cadherin is a cell-cell adhesion molecule, able to anchor epithelial cells through the connection of various catenins to the cytoskeleton [27]. Acquiring this epithelial marker allows cancer cells to lose their metastatic ability, presenting a less aggressive phenotype [27]. N-cadherin is also a glycoprotein that mediates cell-cell adhesion, but it is closely linked to activated metastasis and a poor prognosis in many malignancies [28]. Vimentin, a member of the intermediate filament (IF) family, is a potential prognostic factor of EMT-related proteins [29] and is also viewed as a mesenchymal marker [30]. EMT drives the conversion of cells from an epithelial phenotype to a mesenchymal phenotype and could operate in both physiological and pathological processes such as embryogenesis, wound healing, and carcinoma pathogenesis [31]. In cancer pathogenesis, tumour cells gain a higher migration and invasion ability via EMT. Therefore, our findings indicate that EMT may be an indispensable process for CRIP1-mediated ovarian cancer invasion and metastasis.

Up to this stage, the correlation between CRIP1 expression and cancer-related EMT had been confirmed, but how CRIP1 mediated this process remained unclear. Through KEGG pathway enrichment analysis, we discovered that CRIP1 was significantly enriched in the Wnt/*β*-catenin signaling pathway. This pathway could be stimulated by the binding of the Wnt protein to its receptor called frizzled protein (Frz), which leads to the activation of the downstream cytoplasmic dishevelled protein (Dsh) which blocks the glycogen synthase kinase-3*β*- (GSK-3*β*-) mediated phosphorylation/degradation of *β*-catenin. This allows the accumulation of *β*-catenin and its further translocation into the nucleus for the binding of transcription factors, leading to the subsequent activation of downstream target genes [32]. In this process, *β*-catenin is a core component and functions as an important oncogene in many malignant tumours [32, 33]. Our results confirm that *β*-catenin had relatively high expression in ovarian cancer cells but was downregulated when CRIP1 was knocked down, suggesting that CRIP1 may activate *β*-catenin. Another key molecule is GSK-3*β*, which can act as a key negative regulator and promote *β*-catenin phosphorylation and ubiquitination, leading to subsequent proteasomal degradation. Indeed, in our study, the level of p-GSK-3*β*, but not total GSK-3*β*, was downregulated in ovarian cancer when CRIP1 was silenced. The reason for this may be that the activation of Wnt signaling could lead to the phosphorylation and inactivation of GSK-3*β*, resulting in cytoplasmic GSK-3*β* pool inactivation without influencing total GSK-3*β* levels [34].

Matrix metalloproteinases (MMPs) are important downstream target genes of the Wnt/*β*-catenin signaling pathway [35]. The Wnt/*β*-catenin/MMPs axis acts as an indispensable component of metastasis and EMT in malignant tumours [36]. It has been reported that MMPs can degrade the extracellular matrix (ECM) and basement membrane, which is the first step in cancer invasion and metastasis. Evidence has shown that MMPs are expressed in various cancer types, and the upregulation of MMPs has been associated with tumour progression and invasiveness [37, 38]. As two crucial members of the MMP family, MMP-2 and MMP-9 have also been found to participate in EMT and cancer metastasis [38]. In our study, when CRIP1 was knocked out in ovarian cells, MMP-2 and MMP-9 were significantly downregulated, together with the downregulation of *β*-catenin, N-cadherin, and vimentin, while E-cadherin was upregulated. These results suggest that CRIP1 may activate the Wnt/*β*-catenin signaling pathway, impair cell adhesion, and alter cell migration ability in favour of EMT.

## 5. Conclusion

The present study first demonstrated that CRIP1 may play an oncogenic role in serous epithelial ovarian cancer development and progression, promoting migration and invasion in the OVCAR3 cell line. In particular, this process may be achieved through the Wnt/*β*-catenin signaling pathway by inducing EMT, suggesting that CRIP1 may be an important biomarker for ovarian cancer metastasis and prognosis, as well as an important molecular target for ovarian cancer therapy.

However, this study only validated the effect of CRIP1 on the ovarian cancer OVCAR3 cell line at the cellular level. Furthermore, additional ovarian cancer cell lines, overexpression cell models, and animal models (*in vivo*) are required to confirm the links between CRIP1 expression and ovarian cancer progression and prognosis.

## Figures and Tables

**Table 1:** The specific primer sequences for qRT-PCR are listed.

Gene symbol	Primer category	Primer sequence(5′ to 3′)
CRIP1 (human)	Forward	CCTGCCTGAAGTGCGAGAAAT
	Reverse	CCTTTAGGCCCAAACATGGC

GAPDH (human)	Forward	TGCACCACCAACTGCTTAGC
	Reverse	GGCATGGACTGTGGTCATGAG

**Table 2:** The most important top five genes were displayed through the ranking of 51 target genes by -log_10_ (*P* value).

Symbol	Log_2_ FC	-Log_10_ (*P* value)
TMC4	4.567	131.61083390
RPS6KA1	2.369	80.87942607
CRIP1	4.637	60.73754891
TRPM2	2.111	53.67366414
PLEK2	1.842	49.86327943

**Table 3:** Correlation analysis between CRIP1 protein level and the clinicopathological parameters in 50 cases of serous epithelial ovarian cancer.

Characteristic	Patients (*n*)	CRIP1 expression		*P* value
		High	Low	
Age (years)				0.1452
<50	19	7	12	
≥50	31	18	13	
Tumour diameter				0.6374
<5 cm	5	2	3	
≥5 cm	45	23	22	
Pathological stage				<0.0001
I-II	17	1	16	
III-IV	33	24	9	
Grade				0.0047
G1	10	1	9	
G2-G3	40	24	16	
Lymphatic metastasis				<0.0001
Positive	26	23	3	
Negative	24	2	22	
CA125 (U/ml)				0.3868
<500	30	13	17	
≥500	20	12	8	

## Data Availability

The data used to support the findings of this study are included within the article.
